# Usefulness of electrical activity of the diaphragm to detect intrinsic positive end-expiratory pressure during pressure support ventilation

**DOI:** 10.1186/cc10691

**Published:** 2012-03-20

**Authors:** S Arrigoni, T Mauri, G Bellani, A Pradella, M Turella, V Sala, E Rezoagli, A Pesenti

**Affiliations:** 1 University of Milano-Bicocca, Monza, Italy

## Introduction

Intrinsic positive end-expiratory pressure (PEEPi) may add a substantial workload on respiratory muscles of patients undergoing pressure support ventilation (PSV). This can be reduced with the application of an external positive end-expiratory pressure (PEEPe) [1]. However, an accurate measurement of PEEPi during PSV is challenging [2]. The aim of the present study is to investigate if the use of the electrical activity of diaphragm (EAdi) may yield the detection of PEEPi in patients undergoing PSV. We reasoned that if PEEPi was present the inspiratory airflow would start after EAdi had reached a given value (EAdi-threshold) necessary to generate the muscle pressure overcoming PEEPi.

## Methods

Ten patients with a clinical suspicion of PEEPi undergoing PSV were enrolled. Exclusion criteria were: age <18 years, hemodynamic instability, fever and PaO_2_/FiO_2 _<100 mmHg. All patients were tested during PSV for seven steps of 3 minutes each with increasing PEEPe (2, 4, 6, 8, 10, 12, 14 cmH_2_O). At the end of each step, PEEPi was estimated with an end-expiratory occlusion maneuver. During the study, we continuously recorded airway pressure, flow, volume and EAdi waveforms for off-line analysis. Data were analysed by linear regression and *t *test. *P *< 0.05 was considered statistically significant.

## Results

If PEEPi is present, EAdi-threshold is supposed to gradually decrease together with the raise of PEEPe; thus we divided patients into five responders for whom EAdi-threshold was significantly correlated with PEEPe, as opposed to five nonresponders. In the group of responders we observed significant correlations between the reduction of PEEPi and the increase of PEEPe (*r*^2 ^= 0.86, *P *< 0.01), and between EAdi-threshold and PEEPi at different PEEPe levels (*r*^2 ^= 0.96, *P *< 0.001). In the same group, respiratory rate (RR) decreased (*r*^2 ^= 0.76, *P *= 0.01), tidal volume increased (*r*^2 ^= 0.71, *P *= 0.02) and the peak of EAdi decreased (*r*^2 ^= 0.94, *P *< 0.001) at increasing levels of PEEPe. On the contrary, in the nonresponder group the increase of PEEPe was associated only with an increase of RR (*r*^2 ^= 0.75, *P *= 0.01).

## Conclusion

In five of 10 patients with clinical suspicion of PEEPi, when the PEEPe was increased we observed a decrease of EAdi-threshold, associated with improved respiratory mechanics, suggesting that EAdi-threshold could be a useful indicator for the presence of PEEPi.

## References

[B1] Mancebo J Anesthesiology 2000 93 81 90 10.1097/00000542-200007000-00016 10861149

[B2] Marini JJ Am J Respir Crit Care Med 2011 184 756 762 10.1164/rccm.201102-0226PP 21700908

